# Theoretical Prediction of the Monolayer Hf_2_Br_4_ as Promising Thermoelectric Material

**DOI:** 10.3390/ma15124120

**Published:** 2022-06-09

**Authors:** Qiang Fan, Jianhui Yang, Ning Wang

**Affiliations:** 1 School of New Energy Materials and Chemistry, Leshan Normal University, Leshan 614004, China; fq1893@foxmail.com; 2 School of Mathematics and Physics, Leshan Normal University, Leshan 614004, China; 3 School of Physics, University of Electronic Science and Technology of China, Chengdu 610054, China; ningwang0213@163.com

**Keywords:** monolayer Hf_2_Br_4_, thermal transport, thermoelectric, first-principles calculations

## Abstract

The stability, electronic structure, electric transport, thermal transport and thermoelectric properties of the monolayer Hf_2_Br_4_ are predicted by using first principle calculations combined with Boltzmann transport theory. The dynamic stability of the monolayer Hf_2_Br_4_ is verified by phonon band dispersion, and the thermal stability is revealed by ab initio molecular dynamics simulations. The electronic structure calculation indicates that the monolayer Hf_2_Br_4_ is an indirect band gap semiconductor with a band gap of 1.31 eV. The lattice thermal conductivity of the monolayer Hf_2_Br_4_ is investigated and analyzed on phonon mode level. The calculation results of the electric transport explore the excellent electric transport properties of the monolayer Hf_2_Br_4_. The thermoelectric transport properties as a function of carrier concentration at three different temperatures are calculated. The study indicates that the monolayer Hf_2_Br_4_ can be an alternative, stable two-dimensional material with potential application in the thermoelectric field.

## 1. Introduction

Due to the increasing demand for efficient and clean energy, thermoelectric (TE) materials that can be used in renewable energy installations have attracted extensive attention. According to the Seebeck effect, TE materials can be applied where temperature gradient converts to electrical power and vice versa [1,2]. The conversion efficiency of TE materials is determined by the dimensionless figure of merit, namely the *ZT* value. The *ZT* value at a certain temperature *T* is determined as: $ZT=S^{2}\sigma T/k$. In the equation, *S*, *σ* and k correspond to the Seebeck coefficient, electrical conductivity and thermal conductivity, respectively. Thermal conductivity includes electron ($k_{e}$) and lattice thermal conductivity ($k_{l}$). The $k_{e}$ is related to σ, the relation between them can be described by the Wiedemann–Franz law: $k_{e}=L\sigma T$, in which *L* is the Lorentz constant. S and σ are coupled by the carrier concentration (*n*). The thermoelectric figure of merit *ZT* value can be improved by establishing a combination of enhanced power factor ($PF=S^{2}\sigma$) and low thermal conductivity k. Due to the interdependence of these three inherent parameters (*S*, *σ*, $k_{e}$), optimizing these three parameters to achieve high *ZT* has become a key challenge.

Interestingly, the reduction in the dimensionality has the potential to break aforementioned restrictions to enhance *ZT* value due to the quantum confinement effect [3,4,5]. On the one hand, the quantum confinement effect can improve the density of electronic states (DOS) near the Fermi level and provide a way to decouple *σ* from *S*, resulting in an increased power factor [6,7,8]. The epitaxial CaSi_2_, for example, developed on Si (111) substrates exhibits not only high σ compared to metal but also an extremely large *S* [9]. On the other hand, nanostructing can significantly reduce the lattice thermal conductivity [10,11]. The high-density interfaces provided in nanostructures allow phonons to scatter more efficiently than electrons over an average large free path, resulting in reduced lattice thermal conductivity and maintaining electron transport and electron mobility [12,13,14,15,16]. Thanks to the quantum confinement effect, many studies have found that two-dimensional materials can provide higher TE performance than their bulk counterparts. For example, the PF of SnSeS monolayer is significantly improved several times higher than the bulk analog [17]. Under a moderate hole concentration, the *ZT* value of the Sb_2_Si_2_Te_6_ monolayer reaches 9.62 at 700 K, which is nearly nine times that of the bulk structure [18]. Gupta et al. [19] theoretically predicted that the maximum *ZT* value of SnS monolayer is 1.36 at room temperature, which is almost 33 times higher than the *ZT* of its bulk form.

Recently, Sevil et al. [20] revealed that monolayer Hf_2_Br_4_ is a promising TE material through high-throughput calculation method due to the high electronic fitness function (EFF). However, the electronic and phonon transport mechanism of the monolayer Hf_2_Br_4_ is still puzzling. In this paper, we systematically study the electronic, phonon transport and TE properties of the monolayer Hf_2_Br_4_ with the help of the first principles combined with Boltzmann transport theory.

## 2. Computational Details

All first principal calculations are performed with the help of the Vienna Ab initio Simulation Package (VASP) code [21]. First, we performed the structural optimization using the Perdew–Burke–Ernzerhof (PBE) exchange–correlation functional with the projected augmented wave (PAW) method [22]. The spin–orbit coupling (SOC) is used for the electronic band structure calculations. The cut-off energy for the plane wave basis was set to 500 eV. 15 × 15 × 1 k-meshes in the first Brillouin zone was used. The convergence criteria for energy and force were, respectively, set as 1 × 10^−4^ eV and 1 × 10^−4^ eV/Å. An A~20Å vacuum was applied perpendicular to the layer plane to avoid inaccuracies through interaction with the monolayer. After structural optimization, we performed the electronic band structure calculation, taking into account the Hartree–Fock exchange hybrid functional HSE06 [23]. From the calculated electronic band structure, electronic transport properties were extracted based on the Boltzmann transport theory by employing the BoltzTraP2 code [24], in which relaxation time *τ* is taken as a constant. The denser k-point sampling (31 × 31 × 1) was used in solving the transport equation. To estimate the relaxation time *τ*, we calculated the carrier mobility *μ* near the Fermi level using the deformation potential (DP) theory [25,26,27] through the formula:

(1) $$\mu =\frac{2e\hbar^{3}C}{3k_{B}Tm^{*2}E_{1}^{2}},\;$$

where *e*, ℏ, *k_B_* and *T* are the electron charge, reduced Planck constant, Boltzmann constant and temperature, respectively. The elastic modulus *C*, effective mass m* and DP constant *E*_1_ can be given from the following forms:

(2) $$C=\frac{1}{S_{0}}\left[\partial^{2}E/\partial \delta^{2}\right],$$

(3) $$m^{*}=\hbar^{2}/\left(\partial^{2}\varepsilon /\partial k^{2}\right),$$

(4) $$E_{1}=dE_{edge}/d\delta ,$$

where *E* is the total energy applied uniaxial strain δ. The uniaxial strain δ is defined as $\delta a/a_{0}\;$(Δa is the variation of the lattice parameter, $a_{0}$ is the relaxed lattice parameter), and $S_{0}$ is the area of the optimized surface. ε and k are the band energy and electron wave vector, respectively. $E_{edge}$ denotes the energy of the band edge. The relationship between *τ* and *μ* is related by $\tau =\frac{\mu m^{*}}{e}$.

The lattice thermal conductivity $k_{l}$ is calculated based on the Boltzmann transport equation integrated in ShengBTE code [28] with the second- and third-order interatomic force constants (IFCS) as:

(5) $$k_{l}^{\alpha \beta }=\frac{1}{k_{B}T^{2}\Omega N}\underset{\lambda }{\sum }f_{0}\left(f_{0}+1\right){\left(\hbar \omega_{\lambda }\right)}^{2}v_{\lambda }^{\alpha }v_{\lambda }^{\beta }\tau_{\lambda }^{0},$$

in which *α* and *β* are the Cartesian components of three Cartesian axes (x, y, or z). $\omega_{\lambda }$, $v_{\lambda }$ and $\tau_{\lambda }^{0}$ are the frequency, group velocity and lifetime of phonon mode λ, respectively. Ω, N and $f_{0}$ are the volume of the unit cell, the number of phonon vectors and the Bose–Einstein distribution function, respectively. The boundary scattering is ignored in the calculation. The second-order IFCS were calculated by using the Phonopy code [29] with 7 × 4 × 1 supercells, including 168 atoms. The third-order IFCS were obtained with the help of Thirdorder.py script by constructing a 5 × 3 × 1 supercell with 14th nearest neighbor. Q-grid mesh was set to 40 × 40 × 1 to obtain convergent, accurate lattice thermal conductivity.

## 3. Results and Discussions

### 3.1. Stability and Electronic Structure

**Figure 1:**
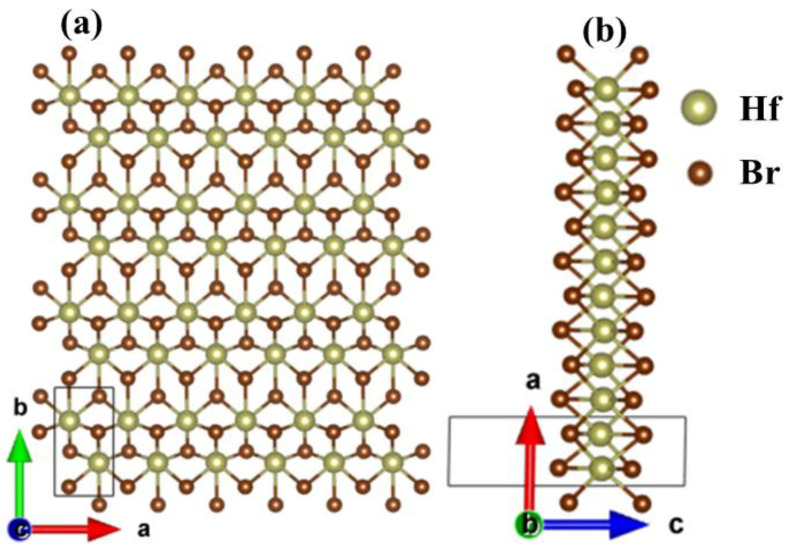
Top (**a**) and side (**b**) views of the 6 × 4 × 1 supercell structure for the monolayer Hf_2_Br_4_. The unit cell is delimited by solid line.

Figure 1 shows the optimized structure of the monolayer Hf_2_Br_4_. The monolayer Hf_2_Br_4_ is a monoclinic structure with P2_1_/m space group, Hf^2+^ and six nearest neighbors Br^1−^ form octahedral structure. There are six atoms in the unit cell, including two Hf^2+^ and four Br^1−^. The out-plane is along the c axis. From Figure 1b it can be seen that the monolayer Hf_2_Br_4_ is composed of three atom layers. The Hf^2+^ layer is sandwiched between two Br^1-^ layers. The optimized lattice constants are a = 3.43 Å and b = 6.41 Å, which are consistent with the previous results (a = 3.43 Å, b = 6.40 Å) obtained by using the first principle PBE functional [20].

**Figure 2:**
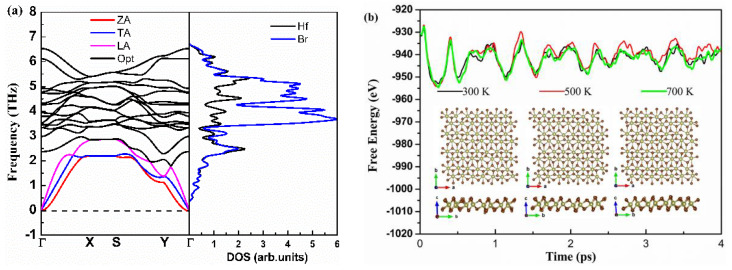
Calculated phonon dispersion and projected density of states (**a**), energy fluctuation with respect to time in AIMD and equilibrium structures at 300, 500 and 700 K (**b**).

First, we investigated the dynamical and thermal stability of monolayer Hf_2_Br_4_. Figure 2a shows phonon dispersion curves obtained from harmonic force constants. Since there are six atoms in the unit cell, there are eighteen phonon modes in phonon dispersion curves. The three acoustic modes include two linear modes (transversal acoustics (TA) and longitudinal acoustic (LA)) in-plane vibrations and one flexural mode (ZA) for out-of-plane vibrations, the other fifteen phonon modes are optical modes. The lowest and highest optical branch frequencies are 1.79 THz and 6.49 THz, respectively. High frequency optical modes are dominated by Br atoms, Hf and Br atoms contribute equally to the low frequency optical and acoustic modes together. The low $k_{l}$ is expected with the large mass difference for Hf and Br atoms [11]. In addition, we noticed that the phonon branch has no imaginary vibrational frequencies, so we can infer that the monolayer Hf_2_Br_4_ is dynamically stable. Moreover, ab initio molecular dynamics (AIMD) simulations of the NPT ensemble with a fixed particle number, pressure and temperature are performed to examine the thermal stability of the monolayer Hf_2_Br_4_ based on the 7 × 4 × 1 supercell. The fluctuation of total energy and the evolution of the structure of the simulation process at 300, 500 and 700 K are shown in Figure 2b. As shown in Figure 2b, the monolayer Hf_2_Br_4_ structure remains crystalline from 300 to 700 K, indicating the thermal stability of the monolayer Hf_2_Br_4_ at the three temperatures. The total energy change per atom is less than 0.5 eV, which is in the acceptable range in such AIMD simulations [30,31]. 

**Figure 3:**
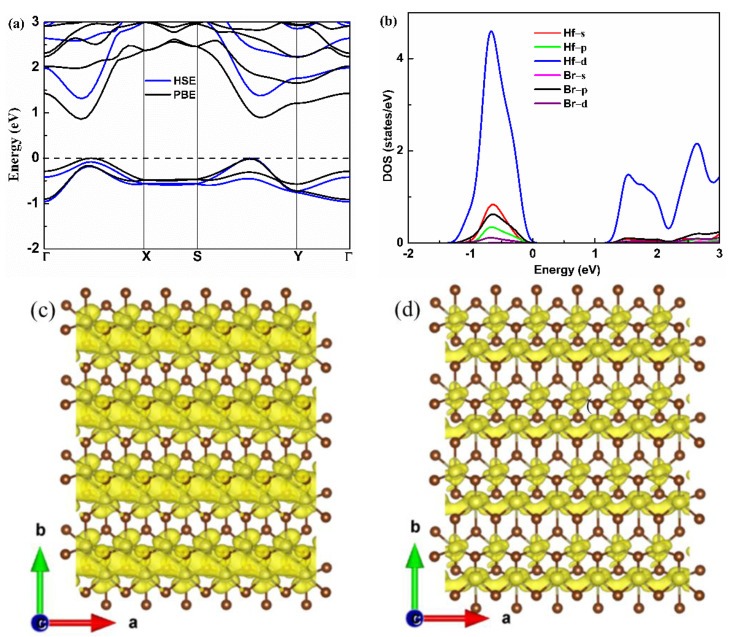
Electronic band structure (**a**), partial DOS (**b**), band decomposition charge density of VBM and CBM (**c**,**d**) for the monolayer Hf_2_Br_4_.

The electronic band structure of the monolayer Hf_2_Br_4_ is shown in Figure 3a. The monolayer Hf_2_Br_4_ is semiconductor with a band gap of 0.86 eV based on the PBE result, which is consistent with the previous report [20]. To accurately evaluate the band gap, the HSE06 functional was used. We found that the HSE06 functional did not change the characteristics of the indirect band gap; however, it gave rise to an increase in the band gap to 1.31 eV. The valence band maximum (VBM) resides between the S−Y path and the conduction band minimum (CBM) is located in Γ−X path. We found that there was a second maximum within 0.1 eV of VBM and CBM along the Γ−X path and S−Y path, respectively, which was in the favor of band degeneracy and led to higher effective density of states (DOS) near VBM and CBM as shown in Figure 3b. We noticed that the CBM had a much higher dispersion than the VBM, indicating that the electron along the a-axis had small effective mass, which was beneficial to electron mobility but was not good for the Seebeck coefficient. The partial DOS (PDOS) of the Hf and Br atoms are shown in Figure 3b. Both the VBM and CBM near the Fermi level were mainly contributed by the d orbital of Hf atoms. The high and steep DOS that occurred near VBM was good for the Seebeck coefficient [32]; therefore, we can assume that the p-type monolayer Hf_2_Br_4_ has superior Seebeck coefficient. A similar phenomenon has been reported in some typical TE materials, such as PbSe [33], BiCuSeO [34] and CaMgSi [35]. The band decomposition charge densities of VBM and CBM with a 0.001 isosurface level are shown in Figure 3c,d. At the VBM, the charge density was localized around Hf and Br atoms, and the charge density of Hf atoms was connected in the a-axis. At the CBM, the charge density was localized around the Br atom at the same isosurface level and formed charge channels in the a-axis. The results demonstrate that the conductive pathway of the p-type and n-type monolayer Hf_2_Br_4_ are mainly determined by the Hf atoms. The charge channels in the a-axis indicate that the electrical conductivity along the a-axis is most likely larger than that along b-axis for both n-type and p-type monolayer Hf_2_Br_4_.

### 3.2. Thermal Transport Properties

**Figure 4:**
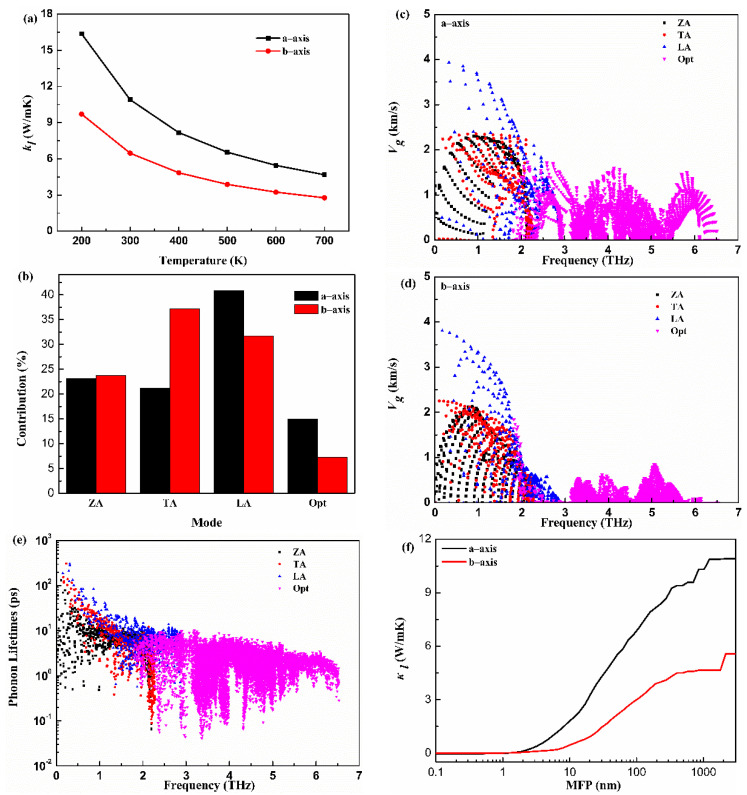
Lattice thermal conductivity ($k_{l}$) as function of temperature (**a**), contributions of phonon modes to lattice thermal conductivity (**b**), frequency-dependence of group velocity along the a-axis and b-axis (**c**,**d**), phone lifetimes (**e**), mean free path (MFP) (**f**) of the monolayer Hf_2_Br_4_.

The lattice thermal conductivity $\left(k_{l}\right)$ is critical to estimate thermoelectric properties. The calculated $k_{l}$ along the a-axis and b-axis versus the temperature (*T*) for the monolayer Hf_2_Br_4_ are given in Figure 4a. The $k_{l}$ decreases with the increase in *T* and demonstrates typical 1/*T* behavior. The $k_{l}$ shows anisotropic features along a different direction. The $k_{l}$ along the a-axis is larger than that along the b-axis at all temperatures. For example, the $k_{l}$ is 10.91 W/mK and 6.47 W/mK along the a-axis and b-axis at 300 K, respectively. The $k_{l}$ of monolayer Hf_2_Br_4_ is lower than that of the reported analog monolayer Hf_2_Cl_4_ [36]. To further explore the physical insights of the thermal transport for the monolayer Hf_2_Br_4_, a further mode level analysis was performed. The $k_{l}$ of the monolayer Hf_2_Br_4_ was further decomposed into different phonon modes. The contributions from the acoustic and optical phonon modes along the a-axis and b-axis at room temperature to the corresponding total $k_{l}$ of the monolayer Hf_2_Br_4_ are shown in Figure 4b. It can be seen that $k_{l}$ is dominated by three acoustic phonon modes. Although the contribution of optical phonon modes is minor, it cannot be ignored, especially along the a-axis. The contribution of 15 optical modes to the total $k_{l}$ along the a-axis and b-axis are 14.9% and 7.2%, respectively. Among the three acoustic modes, the ZA mode contributions to the total $k_{l}$ along the a-axis and b-axis are similar. In order to provide more information about the mechanism of phonon thermal transport, the group velocities (v) and relaxation time (τ) as a function of frequency are illustrated in Figure 4c–e. From the view of the frequency dependence of group velocity, the group velocity of LA mode is larger than that of ZA and TA modes in a-axis and b-axis. In addition, the optical modes along the a-axis show greater group velocity than that along the b-axis. The different group velocities and contributions of phonon models on the a-axis and b-axis led to the anisotropy of thermal conductivity. It is obvious that the low-frequency acoustic modes show longer phonon relaxation times than those of optical modes. Most phonon relaxation times of acoustic modes are in the order of a few to ten picoseconds. Specially, the relaxation times of the acoustic modes with long wavelength are more than 100 picoseconds. The longer relaxation times of acoustic modes account for the dominant role in lattice thermal conductivity. The cumulative $k_{l}$ with respect to the phonon mean free path (MFP) of the monolayer Hf_2_Br_4_ at 300 K is plotted in Figure 4f to discuss the size effect on the phonon transport. We can see that the phonon MFP of monolayer Hf_2_Br_4_ ranges from a few nanometers to nearly 1000 manometers. The phonons with low MFP contribute significantly to the total thermal conductivity. We find that phonons with MFP below 100 nm contribute 63% and 47% to the total $k_{l}$ along the a-axis and b-axis, respectively.

### 3.3. Electronic Transport Properties

**Figure 5:**
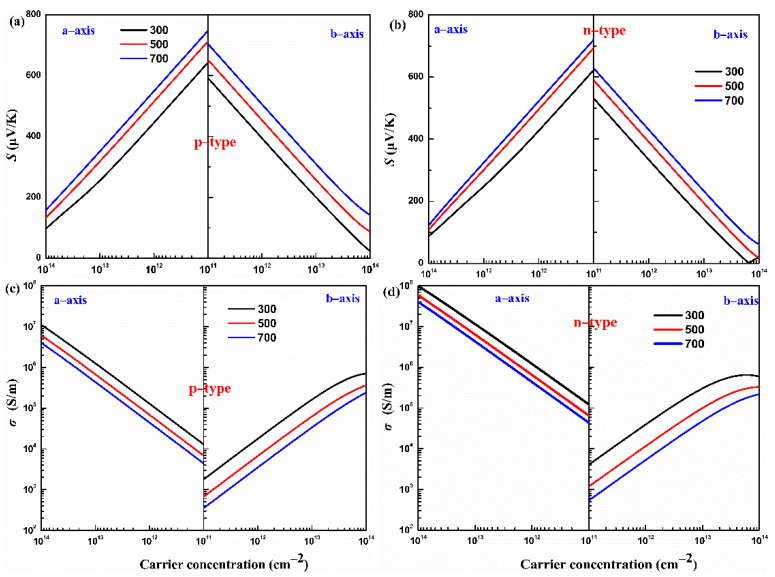
The calculated Seebeck coefficient (*S*) (**a**,**b**) and electrical conductivity (*σ*) (**c**,**d**) of the monolayer Hf_2_Br_4_ at various temperatures (300, 500, and 700 K, respectively).

The Seebeck coefficients of p- and n-type monolayers Hf_2_Br_4_ along the a-axis and b-axis with carrier concentration dependence at 300, 500 and 700 K are shown in Figure 5a,b. For a specific temperature, the absolute value of the Seebeck coefficient (|*S*|) decreases with the carrier concentration from 1 × 10^11^ to 1 × 10^14^ cm^−2^ along the a-axis and b-axis for both the p- and n-types. In addition, at a constant carrier concentration, the |*S*| increases with increasing temperature from 300 K to 700 K along the a-axis and b-axis for both the p- and n-types. The |*S*| is proportional to the temperature but inversely proportional to the carrier concentration, which can be easily understood from $\left|S\right|\propto Tn^{-2/3}$ [37]. We find that the |*S*| of p-type the monolayer Hf_2_Br_4_ is superior to that of n-type at the given temperature and carrier concentration, which can be predicted under the following complied equation [38]:

(6) $$\left|S\right|=\frac{k_{B}}{e}\left[ln\left(\frac{N}{n}\right)+2.5-r\right],$$

where $N,\;n,\;r,\;k_{B}$ and e are effective densities of states near the Fermi level, carrier concentration, scattering parameter, Boltzmann’s constant and electron charge, respectively. The larger slope of the effective state density in VBM, as shown in Figure 3b, results in a larger Seebeck coefficient in the p-type monolayer Hf_2_Br_4_. Moreover, the Seebeck coefficient exhibits anisotropy along the a-axis and b-axis for both p- and n-types. For example, at 700 K, 1 × 10^11^ cm^−2^, the |*S*| of the n-type monolayer Hf_2_Br_4_ are 718 μV/K and 626 μV/K along the a-axis and b-axis, respectively.

Based on Boltzmann transport equation, the electrical conductivity to relaxation time ratio (*σ*/*τ*) is obtained. Generally, the relaxation time (*τ*) at energy *E* and scattering parameter (*r*) is related by power function $\tau \left(E\right)=\tau_{0}{\left(E-E_{0}\right)}^{r}$, in which $E_{0}$ and $\tau_{0}$ are the ground state energy and the corresponding scattering constant [32]. However, various scatters, such as acoustic and optical phonons, interfaces and other carriers, make the relaxation time difficult to determine. In this work, the relaxation time is uncovered based on the DP theory. This approximation method has been widely used to predict the τ for monolayer materials [39,40,41]. The calculated deformation potential constant $E_{1}$, elastic constant *C* and effective mass m* and the corresponding *τ* at 300 K for the monolayer Hf_2_Br_4_ are listed in Table 1. With the help of the calculated relaxation time, the electrical conductivity of the p- and n-types of monolayer Hf_2_Br_4_ along the a-axis and b-axis as a function of carrier concentration at 300, 500 and 700 K is shown in Figure 5c,d. It can be noticed that in the carrier concentration range of 1 × 10^11^ cm^−2^ to 1 × 10^14^ cm^−2^, at a given temperature, the higher the carrier concentration, the higher the electrical conductivity, while the electrical conductivity has the opposite variation with temperature at a given carrier concentration. The electrical conductivity is proportional to carrier concentration and carrier mobility, as explained by the formula: σ=neμ, in which n and μ are the carrier concentration and the carrier mobility, respectively. As the temperature increases, the scattering of carriers increases, which leads to a decrease in carrier mobility. In addition, we find that at a constant temperature and carrier concentration, the electrical conductivity is anisotropic and the electrical conductivity along the a-axis is greater than that along the b-axis no matter for the p-type or n-type monolayer Hf_2_Br_4_. As can be seen from Table 1, the effective mass along the a-axis is much smaller than that along the b-axis, which leads to easier mobility of carriers along the a-axis. On the other hand, charge channels formed in the a-axis are also conducive to carrier mobility, such as the analysis of band decomposition charge density in Figure 3c,d.

### 3.4. Thermoelectric Properties

**Figure 6:**
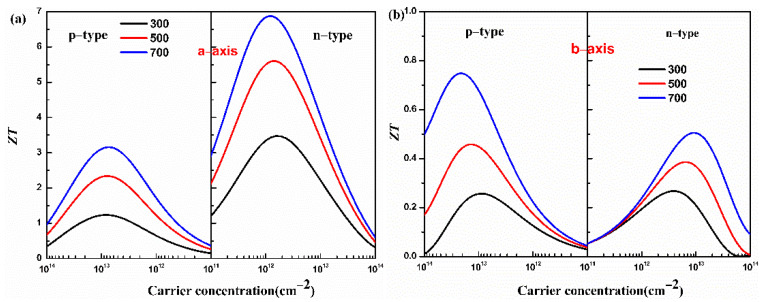
Calculated figure of merit *ZT* of the monolayer Hf_2_Br_4_ along the a-axis (**a**) and b-axis (**b**) for the p- and n-types as a function of carrier concentration at 300, 500 and 700 K.

Combining the properties of electron and phonon transport, the figure of merit *ZT* of the monolayer Hf_2_Br_4_ is predicted. The figure of merit *ZT* of the monolayer Hf_2_Br_4_ as a function of carrier concentration at 300, 500 and 700 K is shown in Figure 6.

From Figure 6, we can see that the monolayer Hf_2_Br_4_ presents an extraordinary thermoelectric performance along the a-axis because of the excellent electrical conductivity along the a-axis. At 700 K, the maximum *ZT* values along the a-axis for the p-type and n-type doping are 3.16 and 6.88 at the optimized carrier concentrations of 7.26 × 10^12^ cm^−2^ and 1.17 × 10^12^ cm^−2^, respectively. At 700 K, along b-axis, the *ZT* values for the p-type and n-type doping reach peaks at carrier concentrations of 1.82 × 10^15^ cm^−2^ and 2.74 × 10^14^ cm^−2^, and the corresponding *ZT* value are 0.75 and 0.30, respectively. These results indicate that the monolayer Hf_2_Br_4_ is a potential thermoelectric material and doping is an effective method to improve the thermoelectric performance.

## 4. Conclusions

In this paper, we systematically calculated the electronic and thermoelectric properties of monolayer Hf_2_Br_4_ using first principles combined with the Boltzmann transport theory. The stability of the monolayer Hf_2_Br_4_ was verified by the phonon dispersion spectrum and AIMD simulations. The calculation of the electronic structure revealed that the monolayer Hf_2_Br_4_ is a semiconductor with an indirect band gap of 1.31 eV and the d orbital of Hf atom is the main contributor for VBM and CBM near the Fermi level. The thermal transport properties calculation showed that the lattice thermal conductivity is dominated by three acoustic phonon modes and exhibits anisotropy caused by the difference in group velocities of low frequency acoustic phonon modes and optical phonon modes. The calculation results of electric transport demonstrate that the monolayer Hf_2_Br_4_ can provide a platform on which relatively high conductivity can coexist with a high Seebeck coefficient. The optimized value of the figure of merit *ZT* along the a-axis under reasonable carrier concentration at 700 K is 3.16 (6.88) for p-type (n-type) doping. In general, the study shows that the monolayer Hf_2_Br_4_ can be an alternative stable two-dimensional material with excellent thermoelectric properties.

## Figures and Tables

**Table 1:** The calculated deformation potential constant $E_{1}$, elastic constant *C* and effective mass m* ($m_{e}$ is the electronic mass) for Hf_2_Br_4_. The relaxation time *τ* at 300 K is listed together.

	Carrier Type	$E_{1\;}(\mathrm{eV})$	*C* (Jm^−2^)	$m*\;(m_{e})$	*τ* (fs)
a-axis	p-type	1.79	81.80	0.79	260.88
	n-type	1.54	81.80	0.22	1265.62
b-axis	p-type	0.56	66.89	1.94	238.63
	n-type	1.08	66.89	1.61	1069.50

## Data Availability

The data presented in this study are available on request from the corresponding author.
